# 2-[(2-Meth­oxy­eth­yl)sulfan­yl]-4-(2-methyl­prop­yl)-6-oxo-1,6-dihydro­pyrimidine-5-carbonitrile

**DOI:** 10.1107/S1600536812013372

**Published:** 2012-04-13

**Authors:** Ali A. El-Emam, Güneş Demirtaş, Necmi Dege, Omar A. Al-Deeb, Nasser R. El-Brollosy

**Affiliations:** a Department of Pharmaceutical Chemistry, College of Pharmacy, King Saud University, 11451 Riyadh, Saudi Arabia; b Ondokuz Mayıs University, Arts and Sciences Faculty, Department of Physics, 55139 Samsun, Turkey

## Abstract

In the title compound, C_12_H_17_N_3_O_2_S, the 4-methyl-2-methyl­sulfanyl-6-oxo-1,6-dihydro­pyrimidine-5-carbonitrile part of the mol­ecule is almost planar (r.m.s deviation = 0.062 Å). In the crystal, mol­ecules form centrosymmetric dimers *via* pairs of N—H⋯O hydrogen bonds.

## Related literature

For related pyrimidine structures, see: Yan *et al.* (2011 ▶); El-Brollosy *et al.* (2011 ▶); Nasir *et al.* (2010 ▶); Tiekink (1989 ▶); Al-Deeb *et al.* (2012 ▶); Durkaya *et al.* (2011 ▶).
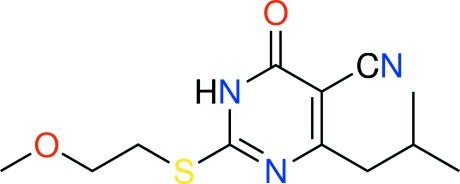


## Experimental

### 

#### Crystal data

C_12_H_17_N_3_O_2_S
*M* *_r_* = 267.35
Triclinic, 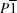

*a* = 5.0379 (5) Å
*b* = 10.5453 (10) Å
*c* = 13.3936 (13) Å
α = 85.274 (8)°
β = 82.170 (8)°
γ = 83.034 (8)°
*V* = 698.14 (12) Å^3^
*Z* = 2
Mo *K*α radiation
μ = 0.23 mm^−1^
*T* = 296 K
0.68 × 0.47 × 0.15 mm

#### Data collection

Stoe IPDS 2 diffractometer
Absorption correction: integration (*X-RED32*; Stoe & Cie, 2002 ▶) *T*_min_ = 0.859, *T*_max_ = 0.966
6663 measured reflections
2725 independent reflections
2090 reflections with *I* > 2σ(*I*)
*R*_int_ = 0.062

#### Refinement

*R*[*F* ^2^ > 2σ(*F* ^2^)] = 0.044
*wR*(*F* ^2^) = 0.126
*S* = 1.02
2725 reflections
164 parameters
H-atom parameters constrained
Δρ_max_ = 0.28 e Å^−3^
Δρ_min_ = −0.22 e Å^−3^

### 

Data collection: *X-AREA* (Stoe & Cie, 2002 ▶); cell refinement: *X-AREA*; data reduction: *X-RED32* (Stoe & Cie, 2002 ▶); program(s) used to solve structure: *WinGX* (Farrugia, 1997 ▶) and *SHELXS97* (Sheldrick, 2008 ▶); program(s) used to refine structure: *SHELXL97* (Sheldrick, 2008 ▶); molecular graphics: *ORTEP-3 for Windows* (Farrugia, 1997 ▶); software used to prepare material for publication: *WinGX* (Farrugia, 1999 ▶) and *PLATON* (Spek, 2009 ▶).

## Supplementary Material

Crystal structure: contains datablock(s) I, global. DOI: 10.1107/S1600536812013372/bt5854sup1.cif

Structure factors: contains datablock(s) I. DOI: 10.1107/S1600536812013372/bt5854Isup2.hkl

Supplementary material file. DOI: 10.1107/S1600536812013372/bt5854Isup3.cml

Additional supplementary materials:  crystallographic information; 3D view; checkCIF report

## Figures and Tables

**Table 1:** Hydrogen-bond geometry (Å, °)

*D*—H⋯*A*	*D*—H	H⋯*A*	*D*⋯*A*	*D*—H⋯*A*
N1—H1⋯O1^i^	0.86	1.89	2.747 (2)	175

Symmetry code: (i) 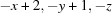.

**Fig. 1.**
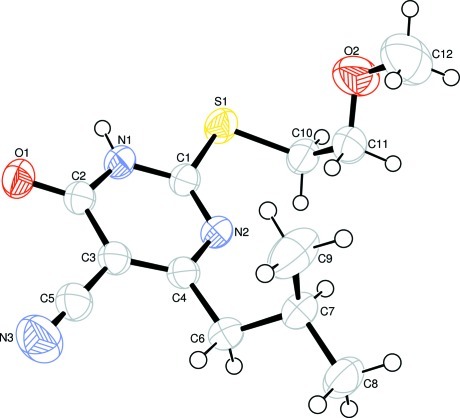
The asymmetric unit of the title compound, showing the atomic numbering scheme. Displacement ellipsoids are drawn at the 50% probability level.

## supplementary crystallographic information

### Comment

In continuation to our work on the chemical and pharmacological properties of pyrimidine derivatives, we synthesized the title compound as a potential chemotherapeutic agent. The 4-methyl-2-(methylthio)-6-oxo-1,6-dihydropyrimidine-5-carbonitrile part of the molecule is almost planar. Some pyrimidine structures have been described in the literature (Yan *et al.*, 2011; El-Brollosy *et al.*, 2011; Nasir *et al.*, 2010; Tiekink 1989; Al-Deeb *et al.*, 2012; Durkaya *et al.*, 2011) and the bond distances of our crystal structure is comparable with these structures.

In the crystal, the molecules form centrosymmetric dimers via intermolecular N—H···O hydrogen bonds.

### Experimental

To a solution of 6-(2-methylpropyl)-4-oxo-2-sulfanylidene-1,2,3,4-tetrahydropyrimidine-5-carbonitrile (2.09 g, 0.01 mol) in DMF (10 ml), 1-bromo-2-methoxyethane (1.4 g, 0.01 mol) and anhydrous potassium carbonate (1.38 g, 0.01 mol) were added and the mixture was stirred at room temperature for 12 h. Water (15 ml) was then added and the mixture was stirred for further 30 min. The separated solid was filtered, washed with cold water, dried and crystallized from water to yield 1.15 g (43%) of the title compound (C_12_H_17_N_3_O_2_S) as colorless crystals. M.P.: 113–115 *^o^*C. Single crystals suitable for X-ray diffraction were obtained by slow evaporation of a solution of the title compound in EtOH at room temperature. ^1^H NMR (DMSO-d_6_, 500.13 MHz): δ 0.93 (d, 6H, CH_3_, *J* = 7.0 Hz), 2.12–2.15 (m, 1H, CH), 2.53 (d, 2H, C*H*_2_CH, *J* = 7.0 Hz), 3.27 (s, 3H, CH_3_O), 3.53 (t, 2H, CH_2_S, *J* = 6.5 Hz), 3.56 (t, 2H, OC*H*_2_CH_2_, *J* = 6.5 Hz), 13.55 (s, 1H, NH). ^13^C NMR (DMSO-d_6_, 125.76 MHz): δ 22.55 (CH_3_), 27.94 (CH), 30.19 (CH_2_S), 45.30 (*C*H_2_CH), 58.36 (CH_3_O), 70.25 (OCH_2_), 95.97 (C-5), 115.55 (CN), 162.05 (C-6), 166.19 (C=O), 174.50 (C-2).

### Refinement

All H atoms were positioned geometrically [N—H = 0.860 Å and C—H = 0.960 Å, 0.970 Å or 0.980 Å] and treated as riding with *U*_iso_(H)=1.2*U*_eq_(C,N).

### Crystal data

**Table:**

C_12_H_17_N_3_O_2_S	*Z* = 2
*M**_r_* = 267.35	*F*(000) = 284
Triclinic, *P*1	*D*_x_ = 1.272 Mg m^−^^3^
Hall symbol: -P 1	Mo *K*α radiation, λ = 0.71073 Å
*a* = 5.0379 (5) Å	Cell parameters from 9417 reflections
*b* = 10.5453 (10) Å	θ = 3.1–27.9°
*c* = 13.3936 (13) Å	µ = 0.23 mm^−^^1^
α = 85.274 (8)°	*T* = 296 K
β = 82.170 (8)°	Prism, colorless
γ = 83.034 (8)°	0.68 × 0.47 × 0.15 mm
*V* = 698.14 (12) Å^3^	

### Data collection

**Table:**

Stoe IPDS 2 diffractometer	2725 independent reflections
Radiation source: fine-focus sealed tube	2090 reflections with *I* > 2σ(*I*)
Graphite monochromator	*R*_int_ = 0.062
rotation method scans	θ_max_ = 26.0°, θ_min_ = 3.1°
Absorption correction: integration (*X-RED32*; Stoe & Cie, 2002)	*h* = −6→6
*T*_min_ = 0.859, *T*_max_ = 0.966	*k* = −12→12
6663 measured reflections	*l* = −16→16

### Refinement

**Table:**

Refinement on *F*^2^	Secondary atom site location: difference Fourier map
Least-squares matrix: full	Hydrogen site location: inferred from neighbouring sites
*R*[*F*^2^ > 2σ(*F*^2^)] = 0.044	H-atom parameters constrained
*wR*(*F*^2^) = 0.126	*w* = 1/[σ^2^(*F*_o_^2^) + (0.0743*P*)^2^] where *P* = (*F*_o_^2^ + 2*F*_c_^2^)/3
*S* = 1.02	(Δ/σ)_max_ < 0.001
2725 reflections	Δρ_max_ = 0.28 e Å^−^^3^
164 parameters	Δρ_min_ = −0.22 e Å^−^^3^
0 restraints	Extinction correction: *SHELXL97* (Sheldrick, 2008), Fc^*^=kFc[1+0.001xFc^2^λ^3^/sin(2θ)]^-1/4^
Primary atom site location: structure-invariant direct methods	Extinction coefficient: 0.059 (9)

### Special details

**Table:**

Geometry. All e.s.d.'s (except the e.s.d. in the dihedral angle between two l.s. planes) are estimated using the full covariance matrix. The cell e.s.d.'s are taken into account individually in the estimation of e.s.d.'s in distances, angles and torsion angles; correlations between e.s.d.'s in cell parameters are only used when they are defined by crystal symmetry. An approximate (isotropic) treatment of cell e.s.d.'s is used for estimating e.s.d.'s involving l.s. planes.
Refinement. Refinement of *F*^2^ against ALL reflections. The weighted *R*-factor *wR* and goodness of fit *S* are based on *F*^2^, conventional *R*-factors *R* are based on *F*, with *F* set to zero for negative *F*^2^. The threshold expression of *F*^2^ > σ(*F*^2^) is used only for calculating *R*-factors(gt) *etc*. and is not relevant to the choice of reflections for refinement. *R*-factors based on *F*^2^ are statistically about twice as large as those based on *F*, and *R*- factors based on ALL data will be even larger.

### Fractional atomic coordinates and isotropic or equivalent isotropic displacement parameters (Å^2^)

**Table:**

	*x*	*y*	*z*	*U*_iso_*/*U*_eq_	
C1	0.6121 (4)	0.32542 (17)	0.13412 (14)	0.0433 (4)	
C2	0.9074 (4)	0.48214 (16)	0.15109 (15)	0.0450 (4)	
C3	0.8266 (4)	0.46228 (16)	0.25758 (15)	0.0442 (4)	
C4	0.6474 (4)	0.37554 (16)	0.29360 (14)	0.0437 (4)	
C5	0.9463 (4)	0.53342 (18)	0.32209 (16)	0.0520 (5)	
C6	0.5689 (4)	0.34539 (18)	0.40400 (14)	0.0495 (5)	
H6A	0.3788	0.3735	0.4212	0.059*	
H6B	0.6694	0.3929	0.4421	0.059*	
C7	0.6216 (4)	0.20252 (19)	0.43492 (15)	0.0479 (4)	
H7	0.5092	0.1569	0.3991	0.057*	
C8	0.5377 (5)	0.1782 (2)	0.54676 (17)	0.0632 (6)	
H8A	0.3491	0.2060	0.5625	0.076*	
H8B	0.6398	0.2249	0.5837	0.076*	
H8C	0.5710	0.0883	0.5652	0.076*	
C9	0.9103 (5)	0.1502 (3)	0.4063 (2)	0.0851 (9)	
H9A	1.0253	0.1946	0.4393	0.102*	
H9B	0.9561	0.1620	0.3344	0.102*	
H9C	0.9339	0.0606	0.4267	0.102*	
C10	0.3129 (4)	0.1261 (2)	0.12329 (17)	0.0566 (5)	
H10A	0.1835	0.1737	0.1707	0.068*	
H10B	0.2118	0.0853	0.0808	0.068*	
C11	0.4729 (5)	0.0231 (2)	0.18197 (18)	0.0646 (6)	
H11A	0.5673	0.0621	0.2278	0.078*	
H11B	0.3517	−0.0319	0.2218	0.078*	
C12	0.7991 (8)	−0.1519 (3)	0.1697 (3)	0.1020 (10)	
H12A	0.6742	−0.2072	0.2050	0.122*	
H12B	0.8951	−0.1190	0.2173	0.122*	
H12C	0.9246	−0.1993	0.1222	0.122*	
N1	0.7874 (3)	0.40967 (14)	0.09326 (12)	0.0468 (4)	
H1	0.8246	0.4179	0.0286	0.056*	
N2	0.5378 (3)	0.30759 (14)	0.23047 (12)	0.0452 (4)	
N3	1.0420 (5)	0.5910 (2)	0.37314 (18)	0.0761 (6)	
O1	1.0717 (3)	0.55469 (13)	0.11167 (11)	0.0575 (4)	
O2	0.6562 (4)	−0.04885 (17)	0.11760 (15)	0.0855 (6)	
S1	0.50676 (11)	0.23771 (5)	0.04486 (4)	0.0547 (2)	

### Atomic displacement parameters (Å^2^)

**Table:**

	*U*^11^	*U*^22^	*U*^33^	*U*^12^	*U*^13^	*U*^23^
C1	0.0492 (9)	0.0408 (9)	0.0385 (10)	−0.0040 (7)	−0.0042 (8)	0.0019 (7)
C2	0.0564 (10)	0.0373 (9)	0.0396 (10)	−0.0048 (7)	−0.0018 (8)	0.0007 (7)
C3	0.0553 (10)	0.0365 (9)	0.0393 (10)	−0.0011 (7)	−0.0048 (8)	−0.0003 (7)
C4	0.0518 (10)	0.0375 (8)	0.0388 (10)	0.0004 (7)	−0.0015 (8)	−0.0005 (7)
C5	0.0637 (12)	0.0444 (10)	0.0473 (11)	−0.0056 (8)	−0.0067 (9)	−0.0014 (9)
C6	0.0616 (11)	0.0483 (10)	0.0365 (10)	−0.0073 (8)	0.0017 (8)	−0.0027 (8)
C7	0.0479 (10)	0.0544 (10)	0.0402 (10)	−0.0077 (8)	−0.0033 (8)	0.0033 (8)
C8	0.0734 (14)	0.0715 (14)	0.0423 (12)	−0.0143 (11)	0.0004 (10)	0.0074 (10)
C9	0.0608 (14)	0.103 (2)	0.0744 (18)	0.0151 (13)	0.0095 (12)	0.0300 (16)
C10	0.0606 (12)	0.0580 (12)	0.0512 (12)	−0.0167 (9)	0.0002 (9)	−0.0007 (9)
C11	0.0896 (16)	0.0523 (11)	0.0507 (13)	−0.0130 (11)	−0.0013 (11)	−0.0013 (10)
C12	0.127 (3)	0.0788 (18)	0.096 (3)	0.0192 (17)	−0.029 (2)	−0.0031 (17)
N1	0.0598 (9)	0.0449 (8)	0.0347 (8)	−0.0099 (7)	−0.0015 (7)	0.0027 (6)
N2	0.0537 (8)	0.0448 (8)	0.0357 (8)	−0.0073 (6)	−0.0008 (7)	0.0007 (6)
N3	0.0948 (15)	0.0671 (12)	0.0729 (15)	−0.0169 (11)	−0.0209 (12)	−0.0147 (11)
O1	0.0751 (9)	0.0526 (8)	0.0456 (8)	−0.0238 (7)	0.0009 (7)	0.0019 (6)
O2	0.1202 (15)	0.0712 (11)	0.0588 (11)	0.0131 (10)	−0.0080 (10)	−0.0074 (9)
S1	0.0701 (4)	0.0594 (3)	0.0368 (3)	−0.0201 (2)	−0.0053 (2)	−0.0007 (2)

### Geometric parameters (Å, º)

**Table:**

C1—N2	1.299 (2)	C8—H8B	0.9600
C1—N1	1.359 (2)	C8—H8C	0.9600
C1—S1	1.744 (2)	C9—H9A	0.9600
C2—O1	1.232 (2)	C9—H9B	0.9600
C2—N1	1.374 (2)	C9—H9C	0.9600
C2—C3	1.434 (3)	C10—C11	1.505 (3)
C3—C4	1.376 (3)	C10—S1	1.800 (2)
C3—C5	1.425 (3)	C10—H10A	0.9700
C4—N2	1.363 (2)	C10—H10B	0.9700
C4—C6	1.496 (3)	C11—O2	1.376 (3)
C5—N3	1.139 (3)	C11—H11A	0.9700
C6—C7	1.530 (3)	C11—H11B	0.9700
C6—H6A	0.9700	C12—O2	1.417 (3)
C6—H6B	0.9700	C12—H12A	0.9600
C7—C9	1.502 (3)	C12—H12B	0.9600
C7—C8	1.510 (3)	C12—H12C	0.9600
C7—H7	0.9800	N1—H1	0.8600
C8—H8A	0.9600		
			
N2—C1—N1	123.67 (18)	C7—C9—H9A	109.5
N2—C1—S1	122.84 (14)	C7—C9—H9B	109.5
N1—C1—S1	113.43 (14)	H9A—C9—H9B	109.5
O1—C2—N1	120.88 (18)	C7—C9—H9C	109.5
O1—C2—C3	125.26 (18)	H9A—C9—H9C	109.5
N1—C2—C3	113.85 (16)	H9B—C9—H9C	109.5
C4—C3—C5	122.88 (18)	C11—C10—S1	115.59 (16)
C4—C3—C2	120.35 (18)	C11—C10—H10A	108.4
C5—C3—C2	116.75 (17)	S1—C10—H10A	108.4
N2—C4—C3	121.83 (17)	C11—C10—H10B	108.4
N2—C4—C6	115.56 (15)	S1—C10—H10B	108.4
C3—C4—C6	122.56 (18)	H10A—C10—H10B	107.4
N3—C5—C3	179.5 (2)	O2—C11—C10	110.6 (2)
C4—C6—C7	112.75 (16)	O2—C11—H11A	109.5
C4—C6—H6A	109.0	C10—C11—H11A	109.5
C7—C6—H6A	109.0	O2—C11—H11B	109.5
C4—C6—H6B	109.0	C10—C11—H11B	109.5
C7—C6—H6B	109.0	H11A—C11—H11B	108.1
H6A—C6—H6B	107.8	O2—C12—H12A	109.5
C9—C7—C8	110.84 (19)	O2—C12—H12B	109.5
C9—C7—C6	112.29 (18)	H12A—C12—H12B	109.5
C8—C7—C6	110.23 (18)	O2—C12—H12C	109.5
C9—C7—H7	107.8	H12A—C12—H12C	109.5
C8—C7—H7	107.8	H12B—C12—H12C	109.5
C6—C7—H7	107.8	C1—N1—C2	122.62 (16)
C7—C8—H8A	109.5	C1—N1—H1	118.7
C7—C8—H8B	109.5	C2—N1—H1	118.7
H8A—C8—H8B	109.5	C1—N2—C4	117.66 (16)
C7—C8—H8C	109.5	C11—O2—C12	112.2 (2)
H8A—C8—H8C	109.5	C1—S1—C10	102.06 (10)
H8B—C8—H8C	109.5		
			
O1—C2—C3—C4	−177.90 (18)	N2—C1—N1—C2	1.5 (3)
N1—C2—C3—C4	0.9 (3)	S1—C1—N1—C2	−175.81 (14)
O1—C2—C3—C5	0.4 (3)	O1—C2—N1—C1	177.75 (17)
N1—C2—C3—C5	179.25 (15)	C3—C2—N1—C1	−1.1 (3)
C5—C3—C4—N2	−179.28 (17)	N1—C1—N2—C4	−1.5 (3)
C2—C3—C4—N2	−1.1 (3)	S1—C1—N2—C4	175.58 (13)
C5—C3—C4—C6	−1.9 (3)	C3—C4—N2—C1	1.3 (3)
C2—C3—C4—C6	176.28 (17)	C6—C4—N2—C1	−176.22 (16)
N2—C4—C6—C7	53.7 (2)	C10—C11—O2—C12	−176.3 (2)
C3—C4—C6—C7	−123.80 (19)	N2—C1—S1—C10	−3.49 (19)
C4—C6—C7—C9	56.1 (3)	N1—C1—S1—C10	173.88 (14)
C4—C6—C7—C8	−179.82 (18)	C11—C10—S1—C1	−70.78 (19)
S1—C10—C11—O2	−59.3 (2)		

### Hydrogen-bond geometry (Å, º)

**Table:**

*D*—H···*A*	*D*—H	H···*A*	*D*···*A*	*D*—H···*A*
N1—H1···O1^i^	0.86	1.89	2.747 (2)	175

Symmetry code: (i) −*x*+2, −*y*+1, −*z*.
